# Editorial: Imaging, diagnosis, and interventional treatment of congenital heart disease in children

**DOI:** 10.3389/fped.2025.1652879

**Published:** 2025-07-18

**Authors:** Mario Carminati

**Affiliations:** Senior Consultant, Milan, Italy

**Keywords:** congenital cardiac anomaly, stent implantation, atrial septal defects, ventricular septal defects, device

## Introduction

Precise diagnosis and accurate anatomical assessment of congenital heart defects are of paramount importance to optimizing subsequent treatment, whether surgical or catheter-based and achieving proper follow-up (1): echocardiography was the “game changer” for the assessment of congenital heart disease a long time ago (CHD); subsequently enormous improvements were obtained by implementing Doppler information, 3D reconstruction, the transesophageal technique etc.… Magnetic Resonance imaging and Computed Tomography have also become increasingly important; angiography, which was essentially the only diagnostic tool available in the pre-echocardiography era, is still used at present, but in the majority of cases, for interventional purposes.

Echocardiography has become the gold standard for prenatal diagnosis and for the assessment of very complex defects. He et al. described prenatally a unilateral pulmonary artery discontinuity, which was found in association with a Taussig-Bing malformation; the diagnosis was confirmed by autopsy, which was performed at the parents' request to terminate the pregnancy. Another successful prenatal diagnosis of a rare case of right atrial dissection/aneurysm was described by Zhang et al. surgical repair was performed early postnatally because of severe tricuspid regurgitation induced by the atrial aneurysm, with restoration of normal tricuspid function.

It is well known that early and correct prenatal diagnosis of CHD plays an important role in postnatal outcomes, allowing better planning of neonatal care, particularly for complex defects. Accurate prenatal diagnoses, particularly for complex cases, are unlikely to be achieved in all centers; Liastuti et al. showed how Artificial Intelligence (AI) can improve the accuracy of CHD detection through second-trimester fetal prenatal screening, particularly in low-income settings. Despite these encouraging results, more extensive studies are necessary to compare AI algorithms with conventional methods and to include a broader range of patients.

Interventional treatment for many CHDs has been described in a large number of cases involving children, adolescents, and adults. More recently interventional options have been described for the closure of patent ductus in premature babies, as an alternative to pharmacological therapy or surgical ligation; transcatheter closure gained increasing popularity, due to improvements in available devices (e.g., Piccolo and other devices) associated with increased expertise of operators. Chmaisse et al. reported encouraging results in the improvement of pulmonary edema and respiratory status after transcatheter closure of the ductus. Some questions remain open regarding which patients should undergo transcatheter duct closure. Apparently the youngest and smallest patients are likely to benefit most from this procedure. Overall transcatheter closure has become the first-choice treatment in many Centers when duct closure is required in premature babies.

Transcatheter closure of a patent ductus arteriosus (PDA) in older children is a well-established procedure; however, some exceedingly rare cases may occur in clinical practice. Zhang et al. described a very unusual case of infective endocarditis originating from a patent ductus arteriosus that was complicated by the formation of an aneurysm of the pulmonary artery. The therapy adopted was conservative, consisting of Vancomycin + Rifampicin for 6 weeks, with resolution of the infection and disappearance of the vegetation. At 6 months follow-up, the patient underwent transcatheter closure of the duct; on CT exam, the pulmonary artery aneurysm appeared relatively small, without any signs of dissection; therefore it was left alone. There is controversy about the need for surgical repair of the aneurysm. The Authors decided to adopt a conservative approach, limiting the invasive intervention to duct closure to eliminate the high aorto-pulmonary flow, which was the cause of pulmonary artery aneurysm, in association with vessel wall damage due to infection. The follow-up, at least in the medium term, was favorable, according to the authors.

Although surgery and Catheter interventions have improved tremendously the treatment of the majority of CHDs in recent years, we should be aware that patients, even if they have been previously treated in the best possible way, cannot be considered “completely cured” and we have to face possible sequelae. Let us consider the example of Coarctation of the aorta: it is a common congenital heart defect, that can present in isolation or be associated with many other defects. Surgery is considered the first choice of treatment in infancy, while angioplasty and stenting (with bare metal or covered stents) have gained progressively increasing popularity in adolescents and adults. Regardless of the treatment performed, persistent hypertension remains a problem in up to 20% of patients who have undergone repair, particularly when the repair itself is done later in life. Ye et al. stressed the importance of a careful follow-up, using a multiparametric approach to assess the problem of “chronic hypertension after correction of coarctation”: in addition to clinical examination, many other tools should be routinely implemented in patient evaluation: echocardiography, magnetic resonance imaging and/or CT, ambulatory blood pressure monitoring, exercise stress testing… In cases of persistent hypertension, despite adequate removal of anatomic obstruction of the aortic arch, pharmacological treatment needs to be considered: Beta-blockers and ACE inhibitors are most commonly used, as are calcium-channel blockers or diuretics in cases of resistant hypertension. Although universally accepted treatment guidelines in this group of patients are lacking, each center is committed to following up with these patients in order to prevent hypertension-induced progressive atherosclerosis and major adverse events.
